# Exploring designability of electrostatic complementarity at an antigen-antibody interface directed by mutagenesis, biophysical analysis, and molecular dynamics simulations

**DOI:** 10.1038/s41598-019-40461-5

**Published:** 2019-03-14

**Authors:** Kouhei Yoshida, Daisuke Kuroda, Masato Kiyoshi, Makoto Nakakido, Satoru Nagatoishi, Shinji Soga, Hiroki Shirai, Kouhei Tsumoto

**Affiliations:** 1 Department of Bioengineering, School of Engineering, The University of Tokyo, 7-3-1, Hongo, Bunkyo-ku, Tokyo 113-8656 Japan; 2 Medical Device Development and Regulation Research Center, School of Engineering, The University of Tokyo, 7-3-1, Hongo, Bunkyo-ku, Tokyo 113-8656 Japan; 3 The Institute of Medical Science, The University of Tokyo, 4-6-1, Shirokanedai, Minato-ku, Tokyo 108-8639 Japan; 4 Modality Research Laboratories, Astellas Pharma Inc., 21, Miyukigaoka, Tsukuba-shi, Ibaraki 305-8585 Japan

## Abstract

Antibodies protect organisms from a huge variety of foreign antigens. Antibody diversity originates from both genetic and structural levels. Antigen recognition relies on complementarity between antigen-antibody interfaces. Recent methodological advances in structural biology and the accompanying rapid increase of the number of crystal structures of proteins have enabled atomic-level manipulation of protein structures to effect alterations in function. In this study, we explored the designability of electrostatic complementarity at an antigen-antibody interface on the basis of a crystal structure of the complex. We designed several variants with altered charged residues at the interface and characterized the designed variants by surface plasmon resonance, circular dichroism, differential scanning calorimetry, and molecular dynamics simulations. Both successes and failures of the structure-based design are discussed. The variants that compensate electrostatic interactions can restore the interface complementarity, enabling the cognate antigen-antibody binding. Retrospectively, we also show that these mutational effects could be predicted by the simulations. Our study demonstrates the importance of charged residues on the physical properties of this antigen-antibody interaction and suggests that computational approaches can facilitate design of antibodies that recognize a weakly immunogenic antigen.

## Introduction

Although antigens far outnumber antibody genes, sufficient antibody diversity results from genetic and structural variation^1^. During B cell development, V(D)J recombination, somatic mutations, and somatic insertions and deletions result in an antibody repertoire that can recognize the enormous number of antigens encountered over a lifetime with excellent specificity^2^. Antibody specificity is determined by the antigen binding site called the complementarity determining region (CDR) and antigen recognition is governed by several types of interactions: hydrogen bonds, electrostatic interactions, van der Waals forces, and hydrophobic interactions^3–5^. As the name suggests, complementarity is a key to antigen recognition. Previous works surveyed complementarity of protein-protein interactions in terms of shape, hydrophobicity, and electrostatics^6–11^. Among them, electrostatic interactions can be controlled by manipulating charged amino acid residues on the interaction surface. For example, generation of charge repulsion at a protein-protein interface would weaken the interaction whereas generation of charge attractions would strengthen the interaction.

Electrostatic force has been harnessed in antibody engineering^12–16^. Starting from crystal structures of antigen-antibody complexes, Lippow *et al*. exploited Poisson–Boltzmann electrostatics to improve binding affinity, showing a 100-fold improvement in affinity over the wild-type antibody^12^. Liu and coworkers demonstrated that substitution of multiple surface residues with charged amino acids, a technique they called supercharging, increased thermal resistance in GFP^13^. Miklos *et al*. subsequently designed thermostable antibodies based on a supercharging strategy^14^; they demonstrated that the designed antibodies had better thermal stability and binding affinity than the parent antibodies even though the altered positions were not in the CDRs but in the framework regions (FRs). The improved affinity was due to a faster on-rate as well as a slower off-rate as measured by surface plasmon resonance (SPR). Kiyoshi *et al*. also started with a crystal structure of an antibody–antigen complex and improved the affinity of the interaction using a computational saturation mutagenesis approach^15^. These researchers identified several affinity-enhancing mutations that were located at the periphery of the interface; interestingly, all were mutated to charged residues. Similarly, Fukunaga and Tsumoto improved the affinity of an anti-human cardiac troponin I antibody by substitution of multiple amino acids in FRs with charged residues^16^. These studies indicate that manipulating electrostatic interactions can improve physicochemical properties of antigen-antibody interactions. To achieve this approach, it is important to understand the physicochemical principles behind the electrostatic complementarity of antigen-antibody interactions.

Molecular dynamics (MD) simulations can reveal the effects of amino acid mutations on a protein structure^17–19^. Wong *et al*. investigated how mutations rigidified CDRs utilizing MD simulations and found that simulations could be useful in designing mutations that resulted in hydrogen bonding and tight packing of side chains^17^. Corrada *et al*. examined correlations between structures of mutated antibodies and affinities for the antigens on the basis of computational and experimental studies, respectively, and proposed a possible mechanism of rigidification^18^. Clark *et al*. attempted direct calculation of relative antigen-antibody binding affinities using a free energy perturbation protocol and replica exchange solute tempering method and was able to predict the effect of alanine mutations on the relative binding affinities^19^. These analyses indicate that MD simulations can reveal dynamic influences that cannot be detected by analysis of a static crystal structure and can be used to predict the effects of protein manipulations on function.

In this study, to clarify the importance of electrostatic interactions and to facilitate design of antigen-antibody interactions, we investigated the effect of surface charge manipulations at an antigen-antibody interface. As a model system, we started with a crystal structure of a complex between neutralizing antibody A6 and the interferon gamma receptor (IFNγR)^20^ available through the Protein Data Bank (PDB)^21^. SPR measurements, circular dichroism (CD) spectra, differential scanning calorimetry (DSC), and MD simulations were performed to quantify kinetics and thermodynamics of the wild-type and the designed antigen-antibody complexes. We also attempted to predict designability of the charged residues solely using a computational approach and discuss a possible strategy to efficiently elicit antibodies against weakly immunogenic antigens by surface charge manipulations.

## Results

### Crystal structure of an antigen-antibody complex guides surface charge manipulations at the interface

**Figure 1:**
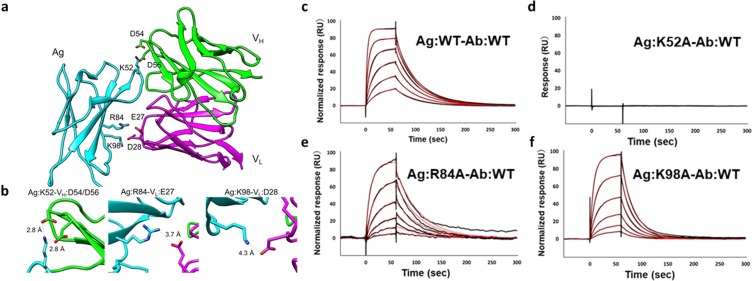
Electrostatic interactions in the IFNγR-A6 complex and the SPR response profiles of the wild-type and the alanine mutant complexes. (**a**) Crystal structure of the antigen (cyan) and antibody (H chain: green, L chain: magenta) complex (from PDB ID: 1JRH, only variable regions are shown). (**b**) Electrostatic interactions at the interface of the wild-type complex. Each distance of electrostatic interaction is displayed as dash lines and was measured based on the crystal structure. Antibody variable regions are numbered based on the Kabat numbering scheme. SPR response profiles of (**c**) **Ag:WT**-**Ab:WT**, (**d**) **Ag:K52A**-**Ab:WT** (**e**) **Ag:R84A**-**Ab:WT**, and (**f**) **Ag:K98A**-**Ab:WT**. The concentrations of the antibody were 1000, 500, 250, 125, 62.5, and 31.25 nM. Raw data are in black; red lines are fitted curves. All fitted data were normalized and assume a 1:1 interaction. Binding kinetics were measured at 298 K.

We chose as our model antigen-antibody system the complex of the extracellular domain D1 of IFNγR and the antibody A6 (Fig. 1a, PDB ID: 1JRH)^20^. Analysis of the crystal structure indicates that three electrostatic interactions contribute to stabilization of the interaction between antigen (Ag) and heavy and light chain variable domains of the antibody (V_H_, and V_L_, respectively) (Fig. 1b). First, K52 of the antigen interacts with the charged β-carboxyl groups of D54 and D56 of the V_H_ domain at distances of 2.8 Å. Second, the γ-carboxyl group of E27 of the V_L_ domain is 3.7 Å from the guanidine group of R84 of the antigen. The main chain of R84 forms part of a β-sheet, and head of exposed side chain contributes to the electrostatic interaction. Third, the β-carboxyl group of D28 of the V_L_ is 4.3 Å from the antigen K98 ε-amino group. Both Ag:K98 and V_L_:D28 are exposed to solvent and in the complex interact with each other.

We performed an SPR assay to experimentally quantify the interaction of the wild-type antibody (**Ab:WT**) with the wild-type antigen (**Ag:WT**); the affinity was high with a *K*_D_ of 87 nM (Fig. 1c and Table 1). We first investigated the importance of each charged residue on the antigen side of the interface. To abolish electrostatic interactions, we prepared three alanine mutants of the antigen, **Ag:K52A**, **Ag:R84A**, and **Ag:K98A**. The SPR measurements showed that **Ag:K52A** did not bind detectably to **Ab:WT** (Fig. 1d), suggesting that K52 of the antigen is a hot spot for the interaction with the antibody. **Ag:R84A** and **Ag:K98A** bound less tightly to the **Ab:WT** than did **Ag:WT** (*K*_D_s 280 nM and 440 nM, respectively compared to 87 nM for **Ag:WT**; Fig. 1e–f, and Table 1). Compared to the wild-type antigen-antibody interaction, the off rates (*k*_off_) almost unchanged, whereas the on rates (*k*_on_) were slower by 3 to 4 fold for the mutant antigens, suggesting that antigen side chains R84 and K98 play important roles in the association of the antigen with the antibody, likely due to the long-range electrostatic attraction to E27 and D28 of V_L_, respectively. These results indicate that mutation of a single charge-charge pair is tolerated, whereas electrostatic interactions involving more than three charged residues disrupt binding completely, perhaps because the position of each residue is cooperatively controlled by multipoint binding.

**Table 1:** Kinetic parameters of SPR analyses.

Antigen-Antibody	*k*_on_ (10^5^ M^−1^ · s^−1^)	*k*_off_ (10^−2^ · s^−1^)	*K*_D_ (10^−8^ M)
**Ag:WT-Ab:WT**	**2**.**7**	**2**.**3**	**8**.**7**
**Ag:R84A-Ab:WT**	**0**.**74**	**2**.**1**	**28**
**Ag:K98A-Ab:WT**	**0**.**90**	**3**.**9**	**44**
**Ag:K52A-Ab:WT**	**N/A**		
**Ag:R84E-Ab:WT**	**N/A**		
**Ag:K98D-Ab:WT**	**N/A**		
**Ag:K52D-Ab:WT**	**N/A**		
**Ag:R84E-V**_**L**_ **:E27R**	**2**.**4**	**1**.**7**	**7**.**3**
**Ag:K98D-V**_**L**_ **:D28K**	**2**.**1**	**2**.**0**	**9**.**5**
**Ag:K52D-V**_**H**_ **:D54K/D56K**	**N/A**		

### Charge complementarity recovery restores antigen-antibody binding

**Figure 2:**
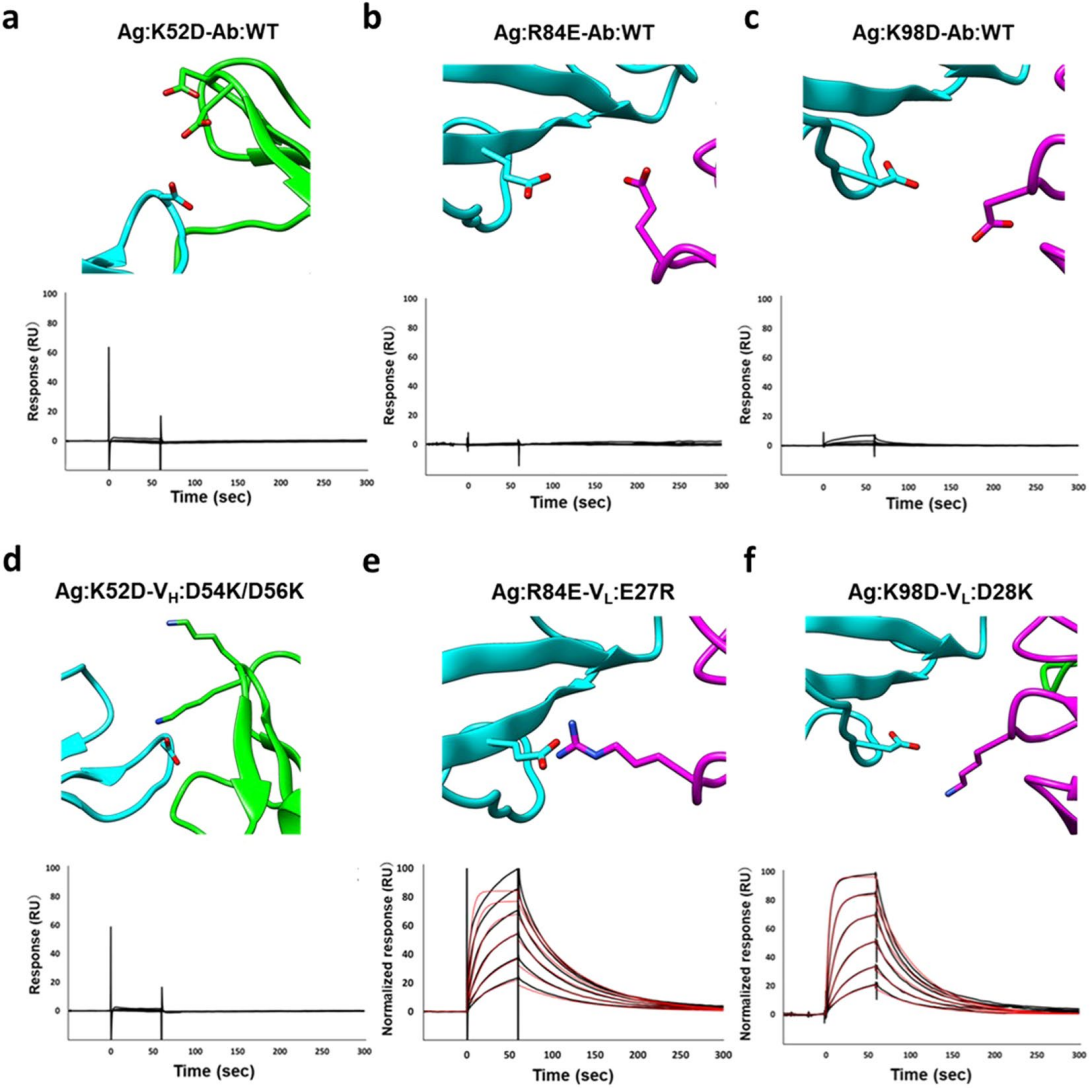
Structures and SPR response profiles of the charge-inverted and charge-exchanged mutants. (**a**) **Ag:K52D-Ab:WT**, (**b**) **Ag:R84E-Ab:WT**, (**c**) **Ag:K98D-Ab:WT**, (**d**) **Ag:K52D-V**_**H**_**:D54K/D56K** (**e**) **Ag:R84E-V**_**L**_**:E27R**, and (**f**) **Ag:K98D-V**_**L**_**:D28K**. Structures of mutants were generated using CHARMM-GUI. The concentrations of the antibody were 1000, 500, 250, 125, 62.5, and 31.25 nM. Raw data are in black; red lines are fitted curves. All fitted data were normalized and assume a 1:1 interaction. Binding kinetics were measured at 298 K.

To explore the designability of electrostatic complementarity at the interface, we further designed three “charge-inverted” antigens (**Ag:K52D**, **Ag:R84E** and **Ag:K98D**; Fig. 2a–c). As expected, the SPR analysis of **Ag:K52D** and **Ag:R84E** showed that these charge-inverted mutants did not bind to the **Ab:WT** (Fig. 2a,b). The other variant, **Ag:K98D**, bound weakly to **Ab:WT** (Fig. 2c). These results suggested that a single electrostatic repulsion can disrupt the antigen-antibody interaction.

To determine whether compensatory changes in the antibody would restore binding, we prepared three “charge-exchanged” antigen-antibody complexes (**Ag:K52D-V**_**H**_**:D54K/D56K**, **Ag:R84E-V**_**L**_**:E27R**, and **Ag:K98D-V**_**L**_**:D28K**; Fig. 2d–f). In the antibody, the charged residues at the interface are complementary. The SPR analysis of the charge-exchanged complexes showed that **Ag:R84E** and **Ag:K98D** interacted with **V**_**L**_**:E27R** and **V**_**L**_**:D28K**, respectively, with binding affinities similar to the wild-type antigen-antibody interaction (*K*_D_s 73 nM and 95 nM, respectively, versus 87 nM for **Ag:WT-Ab:WT**; Fig. 2e,f, and Table 1). Moreover, the on and off rates of the **Ag:R84E-V**_**L**_**:E27R** and **Ag:K98D-V**_**L**_**:D28K** complexes were similar to those of the wild-type antigen-antibody complex.

To verify that loss of binding resulting from charge inverting mutations was caused by electrostatic repulsion, we analyzed the effects of the mutations on the structure and thermostability of antigen and antibody. The CD spectra of the engineered antigens showed that the mutation of R84E changed the secondary structure of the unbound-state of the antigen (Fig. S1a). Given that R84 is partially buried inside the antigen and is part of a β-sheet structure, it was not surprising that the mutation would alter the local structure. The SPR result indicated that the corresponding mutation in the antibody, E27R in the V_L_ domain, restored the binding affinity to near wild-type levels. This suggests that the lack of antibody binding by **Ag:R84E** is mainly caused by electrostatic repulsion.

The individual CD spectra of the compensatory pair **Ag:K98D** and **V**_**L**_**:D28K** were similar to those of the wild-type antigen and antibody (Fig. S1a,b), suggesting that these mutants did not change the conformations, and disruption/restoration of interactions between the two mutants were derived from solely change of electrostatic effect. It is worth noting that the CD spectrum of **Ag:K52D** differed from that of **Ag:WT**; this was not expected as K52 is an exposed residue (Fig. S2). In addition, the corresponding antibody **V**_**H**_**:D54K/D56K** also differed in secondary structure from the wild-type antibody. This suggests that lack of binding of **Ag:K52D** to **Ab:WT** might be due not only to electrostatic repulsion but also structural distortion (Figs 2d and S1).

**Figure 3:**
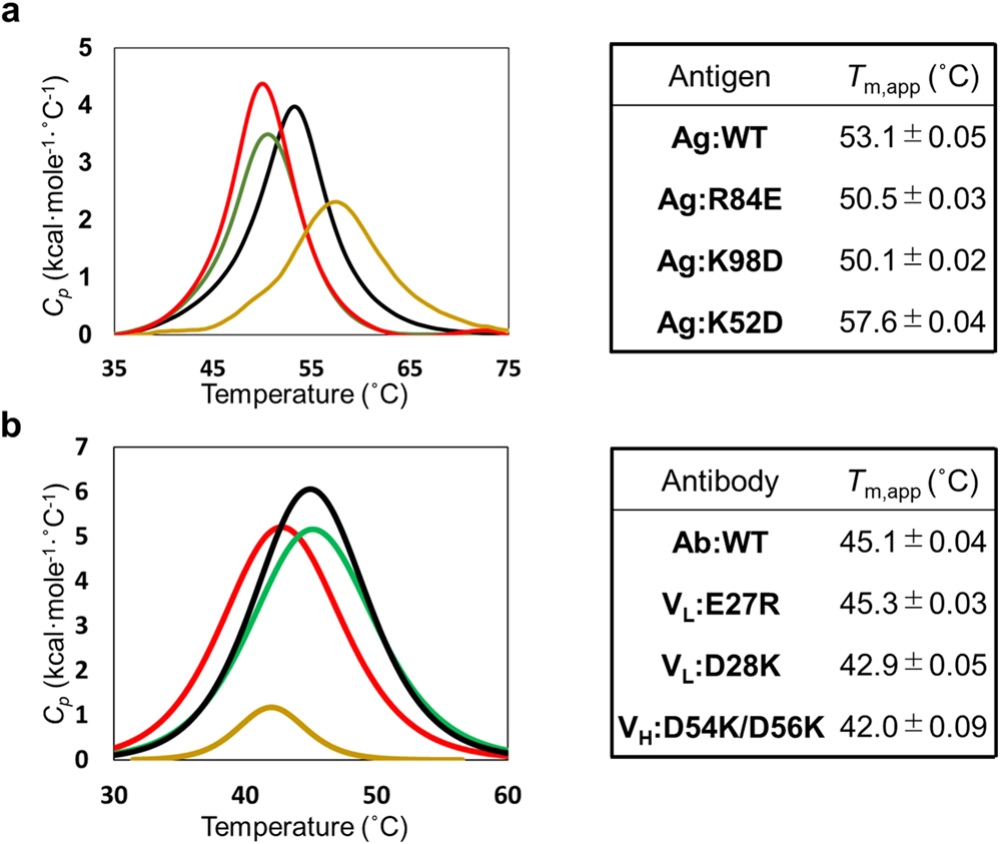
DSC analysis of thermodynamic stabilities of antigen-antibody complexes. (**a**) Melting curves and calculated *T*_m,app_ values of the antigens. **Ag:WT** (black), **Ag:R84E** (green), **Ag:K98D** (red), and **Ag:K52D** (orange). (**b**) Melting curves and calculated *T*_m,app_ values of the antibodies. **Ab:WT** (black), **V**_**L**_**:E27R** (green), **V**_**L**_**:D28K** (red), and **V**_**H**_**:D54K/D56K** (orange). The standard deviation of each *T*_m,app_ value was calculated from the fitting curve.

To further quantify the mutational effects on the structural stability, we carried out DSC assays. The DSC profiles showed that **Ag:K52D** had higher thermal stability than **Ag:WT** antigen; the *T*_m,app_ was 4 °C higher (Fig. 3a). However, the transition peak of the mutant was broad, and a small shoulder was observed, implying that heterogeneous conformations are present. Of antibody mutants, **V**_**H**_**:D54K/D56K** had a significantly smaller Δ*H* and a slightly lower *T*_m,app_ than **Ab:WT** (Fig. 3b). Thus, the DSC results also imply that conformations of **Ag:K52D** and **V**_**H**_**:D54K/D56K** differ from wild-type conformations and thereby disrupting the binding.

### Detailed molecular characterization of mutants reveals the importance of electrostatic interactions

**Figure 4:**
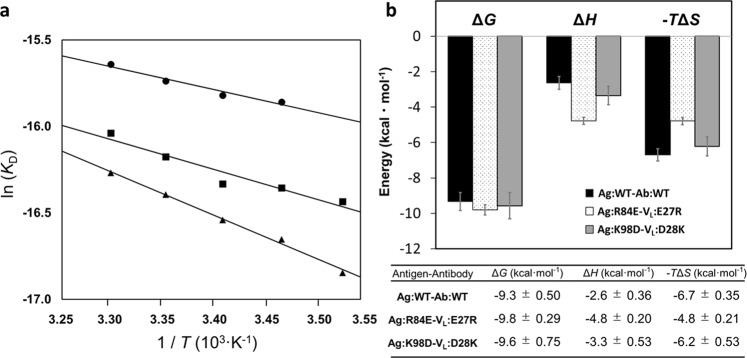
Comparison of thermodynamic profile of antigen-antibody interactions. (**a**) Plots of ln (*K*_D_) vs 1/*T* (K^−1^) for wild-type antigen-antibody (●), **Ag:R84E-V**_**L**_**:E27R** (▲), and **Ag:K98D-V**_**L**_**:D28K** (■). (**b**) Bar graphs of the equilibrium thermodynamic parameters obtained from van’t Hoff analyses. Error bars correspond to the standard deviation. The standard deviations of Δ*H* and −*T*Δ*S* were calculated from the fitting data, respectively, and that of Δ*G* was calculated from Δ*G* = Δ*H* − *T*Δ*S* with error propagation. Values are reported at 298 K.

Next, we evaluated thermodynamics of the interactions for each mutant by SPR as a function of temperatures. The thermodynamic parameters were determined from van’t Hoff plots (Fig. 4a). Although the charge-exchanged mutations were kinetically similar to wild type (Fig. 2e,f, and Table 1), **Ag:R84E-V**_**L**_**:E27R** and **Ag:K98D-V**_**L**_**:D28K** had different thermodynamic profiles (Fig. 4b). Compared to the wild-type antigen-antibody interaction, the **Ag:R84E-V**_**L**_**:E27R** interaction was driven more enthalpically, whereas the balance of enthalpic and entropic contributions to the **Ag:K98D-V**_**L**_**:D28K** interaction were almost same as those of the wild-type complex.

To understand the molecular mechanism underlying the change in thermodynamic parameters due to charge-exchanged mutations we examined the intramolecular environment surrounding each mutated charged residue. The crystal structure of the antigen-antibody complex suggests that the guanidine group of the Ag:R84 side chain has cation-π interactions with surrounding aromatic residues such as Ag:W56 and Ag:Y96. Ag:R84 also forms an electrostatic interaction with Ag:E45 as the distance between the guanidine group of Ag:R84 and γ-carboxyl group of Ag:E45 is 2.9 Å (Fig. S2). Therefore, in addition to the salt bridge with V_L_:E27 of the antibody, Ag:R84 stabilizes the structure of the antigen itself.

**Figure 5:**
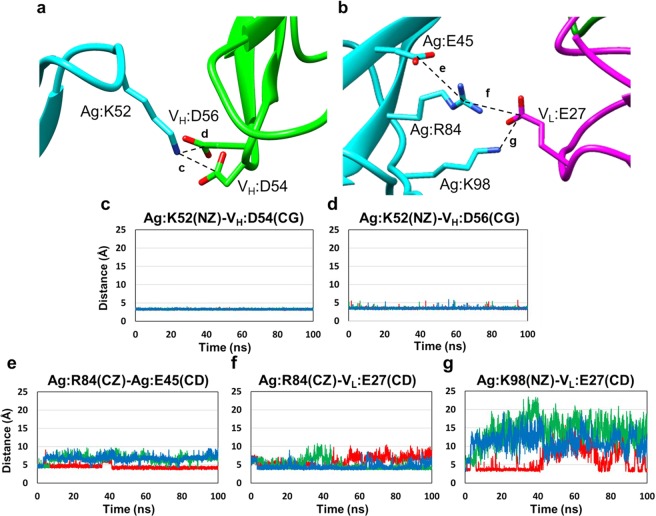
Distances between charged residues that contribute to electrostatic interactions during the MD simulations. (**a**,**b**) Electrostatic interactions at the interface of the wild-type complex. Distances between each atom were displayed as dash lines. Distances between (**c**) Ag:K52 and V_H_:D54, (**d**) Ag:K52 and V_H_:D56, (**e**) Ag:R84 and Ag:E45, (**f**) Ag:R84 and V_L_:E27, and (**g**) Ag:K98 and V_L_:E27. Each distance was measured based on NZ, CG, CZ, and CD atoms of Lys, Asp, Arg, and Glu, respectively. Each 100 ns run was performed three times, indicated in red, green, and blue lines.

To further understand the molecular details of the reorganization of the electrostatic complementarity, we performed multiple 100 ns MD simulations of the wild-type complex as well as **Ag:R84E**-**V**_**L**_**:E27R** and **Ag:K98D**-**V**_**L**_**:D28K** complexes, which had kinetics similar to that of the wild-type complex. We first computed the root mean square deviation (RMSD) of the Cα atoms of the complexes, except for the first and last five residues of each chain, with respect to the reference crystal structure 1JRH (Fig. S3). The RMSD values were quite stable after 20 ns. Therefore, in the analyses below, we did not consider the first 20 ns of the trajectories. First, we confirmed the stable interaction at the predicted hotspot by measurement of the distances between Ag:K52 (NZ atom) and V_H_:D54 and V_H_:D56 (CG atoms). In the **wild-type** complex, the distance between Ag:K52 and V_H_:D54 was within 3.6 Å, which is the distance in the crystal structure, in ~100% of the snapshots in the last 80 ns of each simulation and the distance between Ag:K52 (NZ atom) and V_H_:D56 (CG atom) was within 3.5 Å in 55.3 ± 2.0% of the snapshots (Fig. 5a,c–d). In the crystal structure, Ag:R84 interacts with intramolecular aromatic residues (Ag:W56 and Ag:Y96) and a charged residue (Ag:E45) (Fig. S2). In two out of three simulations, Ag:R84 tended to interact with V_L_:E27 as seen in the crystal structure; this interaction was observed in 77.3% and 83.3% of the snapshots, respectively. In contrast, in the other simulation Ag:R84 tended to form a salt bridge with Ag:E45; these residues were within 4.3 Å, which is the distance between CZ and CD atoms of Arg and Glu, respectively, in the crystal structure, in 48% of the snapshots, and the interaction between Ag:R84 and V_L_:E27 was observed in only 26.7% (Fig. 5b,e,f). Moreover, in this simulation, V_L_:E27 also interacted with Ag:K98, which was spatially adjacent to the Ag:R84. The distance between V_L_:E27 (CD) and Ag:K98 (NZ) was 8.0 Å in the crystal structure, whereas the distance was less than or equal to 4.0 Å in 37.7% of the snapshots (Figs 1a and 5b,g). Thus, in the simulations of the wild-type complex, charge-charge interactions were well established during the simulations.

In the simulations of the **Ag:K98D**-**V**_**L**_**:D28K** complex, as expected, Ag:K98D interacted with V_L_:D28K within 4.0 Å in 62 ± 2% of the snapshots. In addition, Ag:K98D also interacted with Ag:R84 due to the charge attraction, and these residues were within 4.0 Å in 17 ± 5% of the snapshots. These salt bridges were mutually exclusive with the interaction network comprised of Ag:R84, Ag:E45, Ag:W56, and Ag:Y96, which was observed in the crystal structure. The Ag:K98D interaction with Ag:R84 placed Ag:R84 closer to V_L_:E27 (Fig. S4a,b). In the simulations of the **Ag:R84E**-**V**_**L**_**:E27R** complex, Ag:R84E interacted with V_L_:E27R within 4.0 Å in 88 ± 3% of the snapshots. Ag:K98 attracted Ag:R84E, making a cavity near V_H_:E45, V_H_:Y56, and V_H_:W96 (Fig. S4c,d). V_L_:E27R was located in this cavity and formed interactions electrostatic or cation-π interactions with V_H_:E45, V_H_:Y56, and V_H_:W96 (Fig. S4e). Therefore, as with the simulations of the wild-type complex, the charge-charge interactions were observed in the simulations of the two charge-exchanged complexes (**Ag:R84E**-**V**_**L**_**:E27R** and **Ag:K98D**-**V**_**L**_**:D28K**).

These results suggest that although the electrostatic interactions observed in the crystal structure were important in the association of mutant antigen-antibody complexes, other intermolecular and intramolecular interactions that were not obvious from the static crystal structure also play roles in the association: Surrounding residues can compensate for loss of electrostatic interactions as binding is stabilized by the surrounding residues.

## Discussion

Electrostatic interactions are one of the important forces in antigen-antibody recognitions. Previous studies demonstrated that binding affinities of antibodies could be enhanced based on electrostatic interactions^12,15^. Another study suggested that electrostatic interactions at antigen-antibody interfaces contributed to binding specificity and affinity due to loss of conformational flexibility through geometric constraint^22^. In this study, we carefully characterized the electrostatic interactions of neutralizing antibody A6 with its antigen IFNγR as a model system for structure-based design of antigen-antibody complexes. Of three charged residues changed pair-wise, we succeeded in the modulation of electrostatic interactions for two combinations **Ag:R84E-V**_**L**_**:E27R** and **Ag:K98D-V**_**L**_**:D28K**. In contrast, the interactions between Ag:K52 and V_H_:D54/D56 were not restored by the charge-exchanged mutations (Fig. 2d–f). Our biophysical analyses using CD and DSC indicate that **Ag:K52D** and **V**_**H**_**:D54K/D56K** caused structural disruptions that could interfere with complex formation (Figs S1 and 3). All the three residues (Ag:K52, V_H_:D54/D56) are exposed to solvent (Fig. 1b) and appeared not to form intramolecular bonds in the crystal structure. However, the **Ag:K52D** and **V**_**H**_**:D54K/D56K** mutations might have affected the conformation of the antigen and the antibody, respectively. Our results demonstrate the importance of investigating structure and/or thermal stability coupled with interaction analysis when electrostatic mutation-based analyses are conducted.

**Figure 6:**
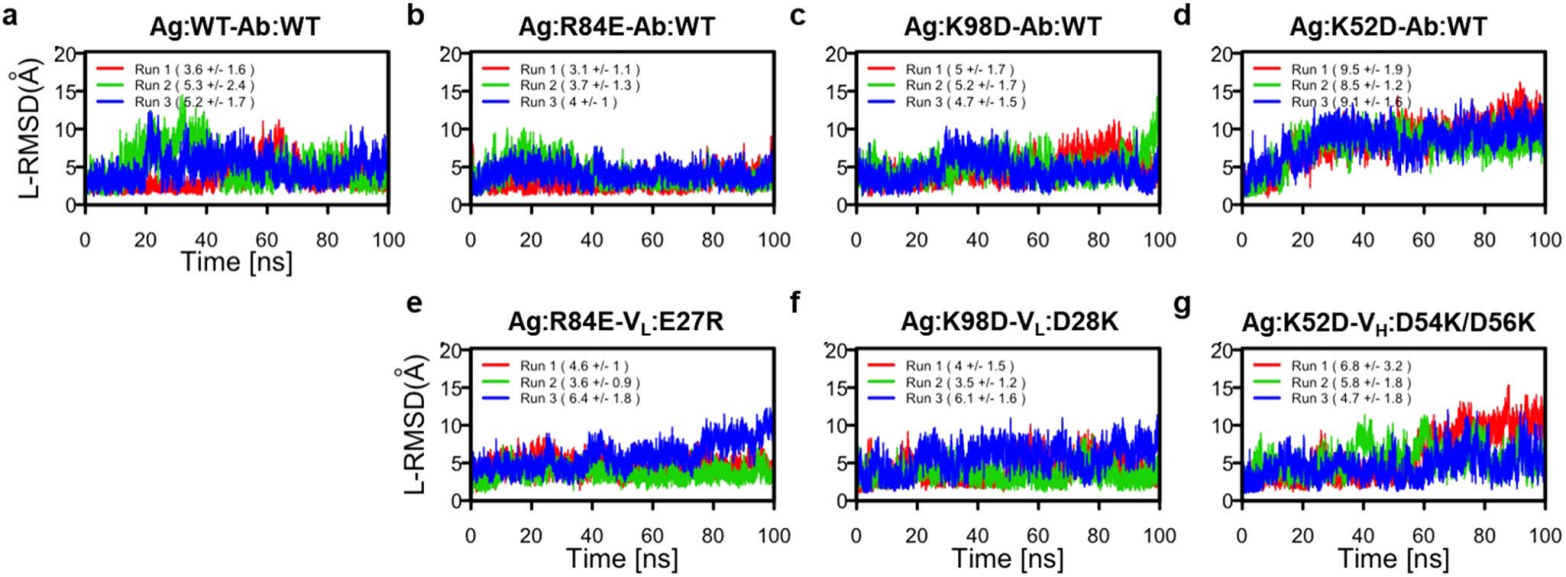
RMSDs of the antigen-antibody interactions between simulations and initial structure. L-RMSD values of (**a**) wild-type antigen-antibody interactions, (**b**–**d**) charge-inverted antigen-antibody interactions, and (**e**–**g**) charge-exchanged antigen-antibody interactions. Each 100 ns run was performed three times, indicated in red, green, and blue lines. Averages and standard deviations of the L-RMSDs are indicated in parentheses.

Our MD simulations were able to illustrate how the charge-exchanged residues of **Ag:R84E-V**_**L**_**:E27R** and **Ag:K98D-V**_**L**_**:D28K** complexes modulate the electrostatic complementarity. It would be difficult to observe charge association and compensation of electrostatic interactions by surrounding residues solely from the static crystal structure and/or *in vitro* measurements. These successes provoked us to perform additional MD simulations to evaluate the predictability of effects of mutations at the antigen-antibody interface. Therefore, in addition to the aforementioned simulation studies for two charge-exchanged variants (**Ag:R84E-V**_**L**_**:E27R** and **Ag:K98D-V**_**L**_**:D28K**), we also conducted MD simulations of three charge-inverted variants **Ag:K52D**-**Ab:WT**, **Ag:R84E**-**Ab:WT**, and **Ag:K98D**-**Ab:WT** as well as the other charge-exchanged variant **Ag:K52D**-**V**_**H**_**:D54K/D56K**. We analyzed the relative orientation between the antigen and antibody by computing RMSDs of the Cα atoms of the antibody after superposing the Cα atoms of the antigen in the trajectories; these values are referred to as ligand-RMSD (L-RMSD)^23^. The L-RMSD of the wild-type complex was stable throughout the trajectory, suggesting that the simulation resulted in the stable conformation of the antigen-antibody complex (Fig. 6a). Our SPR assays suggested that **Ag:R84E-Ab:WT** and **Ag:K98D-Ab:WT** complexes were significantly less stable than the **Ag:WT-Ab:WT** complex (Figs 1c and 2b,c); however, the L-RMSD plots for these two complexes were similar to that for the wild-type complex (Fig. 6b,c). For the **Ag:K52D-Ab:WT** complex, L-RMSD values gradually increased, and the complex appeared to dissociate (Fig. 6d), suggesting that the simulation did appropriately reflect the weakened interaction caused by charge inversion (Fig. 2a). As expected, the L-RMSD values of two charge-exchanged variants **Ag:R84E**-**V**_**L**_**:E27R** and **Ag:K98D**-**V**_**L**_**:D28K** were similar to that of the wild-type complex (Fig. 6e,f) in agreement with our SPR data. Moreover, in the MD simulations, **Ag:K52D**-**V**_**H**_**:D54K/D56K** did not form a stable complex, as reflected by high L-RMSD values during the trajectories (Fig. 6g), also concordant with our SPR analysis. From these observations, we suggested that L-RMSD from MD trajectories would be an appropriate metric that could be used to assess mutational effects on protein-protein interfaces.

**Figure 7:**
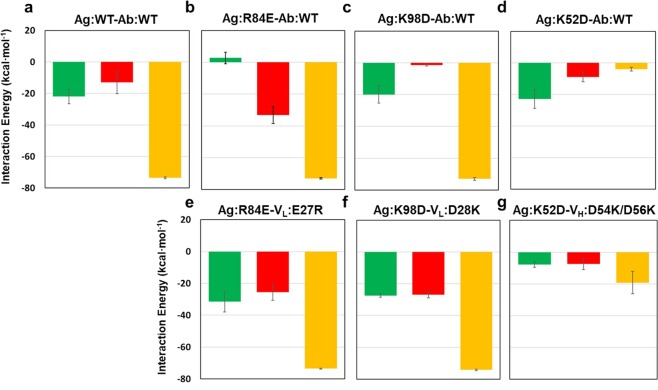
Interaction energy of the antigen-antibody interactions in the simulations. Interaction energies of antigen residues with antibody in (**a**) the wild-type antigen-antibody complex, (**b**–**d**) the charge inverted antigen-antibody complexes, and (**e**–**g**) charge-exchanged antigen-antibody complexes. Energies of the residues at the position of 84, 98, and 52 are shown in green, red, and orange, respectively. Standard errors were indicated by black lines (n = 3).

Furthermore, we also performed interaction energy calculations (van der Waals plus coulomb energy) to the mutated residues in the antigen (K52, R84, and K98), enabling to complement the prediction based on L-RMSD. In the wild-type complex and the two charge-exchanged variant **Ag:R84E**-**V**_**L**_**:E27R** and **Ag:K98D**-**V**_**L**_**:D28K**, which were stable as shown by SPR, Ag:K52 made the largest energy contributions, suggesting that Ag:K52 was a hot spot in the antigen-antibody interaction, in consistent with our SPR and L-RMSD results (Fig. 7). Importantly, the calculation showed significant loss of interaction energy due to each of the three charge-inverted mutations compared to the wild-type complex (Fig. 7a–d). Moreover, whereas the interaction energy was restored by charge-exchange mutation in **Ag:R84E-V**_**L**_**:E27R** and **Ag:K98D-V**_**L**_**:D28K**, the interaction energy was not restored in case of **Ag:K52D**-**V**_**H**_**:D54K/D56K** (Fig. 7e–g). Although the calculations of interaction energies isolated only the enthalpic contribution without considering entropic effects, it showed reasonable correlation with the SPR data. Thus, MD simulations followed by energy calculations could provide important guidance for antigen-antibody manipulation, enabling computer-aided rational antibody design^24^.

In rational antibody engineering for therapeutic and biotechnological purposes^25,26^, antibody elicitation is the important first step. There are several methods for antibody generation but the most widely used method is animal immunization, where an antigen is injected into an animal. Certain antigens of interest are weakly immunogenic likely due to high homology to protein from immunized animal. Several display technologies have been developed to enable generation of antibodies, but acquisition of antibodies with high affinity, specificity, and stability is challenging.

As it has been suggested that only a single mutation can make proteins more immunogenic^27^, we suggest that charge conversion is a possible strategy for generation of antibodies against weakly immunogenic antigens. First, one would modulate the surface charge of the antigen to potentiate immunogenicity. After antibodies are obtained against the charge-modulated antigen, the charged residues in the antibody that interact with modified charged residue in the antigen would be identified and converted to match that of the antibody. Our study suggests that the resultant antibody should interact specifically with original antigen (Fig. S5). In addition, our result showing that charge-inverted antibody did not bind to antigen implies that antibodies generated by immunization with charge-modified antigens will not recognize homologous proteins from host animal.

Finally, although many previous studies in protein engineering have tried to improve binding affinities by introducing electrostatic pairs, binding affinity must be modulated appropriately to retain a desired function^28^. In this study, our designed alanine mutations resulted in the slower *k*_on_ but did not abolish the binding (Fig. 1e–f). The alanine mutation to a charged residue that is involved in a single negative-positive charge pairing might be a promising approach to decrease affinity by three to five fold. Thus, design of electrostatic complementarity, guided by both experiments and molecular simulations is an effective approach for manipulation of antigen-antibody interaction.

In this study, we presented the structure-based design of electrostatic complementarity of an antigen-antibody complex, and the following biophysical analysis showed the success and failure of these designed variants in terms of the recovery of binding capability. We also suggested the availability of MD-based prediction in the design of an antigen-antibody complex. Our results supplied insights that each electrostatic interaction has different role in an antigen-antibody binding process and should be considered in view of structural and dynamic aspects for rational manipulation of electrostatic complementarity.

## Methods

### Expression and purification of antibodies

The single-chain variable fragment (scFv) was constructed from the DNA encoding the Fab of A6; the DNA was chemically synthesized by GeneArt. A (GGGGS)_3_ linker was inserted between the H chain and the L chain, and the sequence was optimized for expression in *E*. *coli*. The A6 scFv construct was expressed in vector pRA2 to result in a hexa-histidine tag at the C terminus. The DNA sequence was confirmed by the dideoxy chain-termination sequencing method.

Cells of *E*. *coli* strain BL21 (DE3) (Novagen) were transformed with the A6 scFv expression vector and grown at 28 °C in LB medium. Protein expression was induced by addition of 0.5 mM isopropyl β-D-1-thiogalactopyranoside when the optical density at 600 nm reached a value of 0.6. Cells were allowed to grow for an additional 16 h at 20 °C. The cells were harvested by centrifugation at 8,000 × g for 10 min, and the pellet thus obtained was resuspended in a solution containing 0.2 M NaCl, 1 mM EDTA, and 50 mM Tris-HCl, pH 8.0 (buffer A). Cells were subsequently lysed by sonication with an ultrasonic cell-disruptor instrument (TOMY); samples were sonicated twice for 20 min each period (Output 7, Duty 50).

The pellet containing the insoluble intracellular components was obtained by centrifugation at 8,000 × g for 30 min. The supernatant was discarded, and the insoluble fraction was resuspended in 1% Triton-X100 (Wako) in buffer A. After centrifugation at 8,000 × g for 15 min, the insoluble fraction was resuspended in water, and centrifuged at 8,000 × g for 15 min; this removed the detergent. The insoluble fraction was resuspended in acetone and centrifuged at 10,000 × g for 30 min to remove lipids. The insoluble fraction was resuspended in a water to remove remaining acetone, and the inclusion body was obtained by centrifugation at 8,000 × g for 15 min. The pellet was solubilized with 6 M guanidine-HCl, 0.4 M NaCl, 20 mM Tris-HCl, pH 8.0 (buffer B) and 5 mM imidazole and incubated overnight at 4 °C. The supernatant collected after centrifugation at 40,000 × g for 30 min was applied to an Ni-NTA agarose (QIAGEN) column equilibrated with buffer B. The column was washed sequentially with 5 and 10 mM imidazole in buffer B. The A6 scFv was eluted with 300 mM imidazole in buffer B.

The purified A6 scFv was refolded using a stepwise dialysis method. The wild-type A6 scFv was diluted to 7.5 µM, and mutants were diluted to 1 µM with buffer B, followed by stepwise dialysis to remove the denaturant. In order to increase the refolding efficiency, 0.4 M Arg-HCl was added to the dialysis solution to minimize protein aggregation when the concentration of guanidine-HCl decreased from 1 M to 0.5 M as previously described^29^. The refolded A6 scFv was concentrated and purified over an Ni-NTA agarose column, and subjected to size exclusion chromatography on a HiLoad 26/600 Superdex 75-pg column (GE Healthcare) equilibrated with 0.4 M NaCl, 1 mM EDTA, and 20 mM Tris-HCl, pH 8.0. The monomer peak fraction was collected.

### Expression and purification of antigen

The DNA encoding the extracellular domain D1 of IFNγR was chemically synthesized by GeneArt with sequence optimized for expression in *E*. *coli*. The IFNγR construct was expressed in vector pET28b to result in a hexa-histidine tag at the C terminus. The DNA sequence was confirmed by sequencing using the dideoxy chain-termination method. Cells of *E*. *coli* strain BL21 (DE3) (Novagen) were transformed with the IFNγR expression vector and were grown at 37 °C in LB. Protein expression was induced by addition of 0.5 mM isopropyl β-D-1-galactopyranoside when the optical density at 600 nm reached a value of 0.6. Cells were allowed to grow for an additional 16 h at 20 °C. The cells were harvested by centrifugation at 8,000 × g for 10 min, and the pellet thus obtained was resuspended in a solution containing 0.5 M NaCl, 5 mM imidazole, and 20 mM Tris-HCl, pH 8.0 (buffer C). Cells were subsequently lysed two 20-min periods of sonication method with an ultrasonic cell-disruptor instrument (TOMY; Output 7, Duty 50).

The supernatant containing the soluble intracellular components was obtained by ultracentrifugation at 40,000 × g for 30 min. The soluble fraction was applied to an Ni-NTA agarose (QIAGEN) column equilibrated with buffer C. The column was washed sequentially with 5 and 10 mM imidazole in buffer C. IFNγR was eluted with 300 mM imidazole in buffer C. The purified IFNγR was subjected to size exclusion chromatography over a HiLoad 26/600 Superdex 75-pg column (GE Healthcare) equilibrated with 0.2 M NaCl, 1 mM EDTA, and 50 mM Tris-HCl, pH 8.0. The monomer peak fraction was collected.

### Kinetic and thermodynamic SPR assays

The interaction between A6 and IFNγR was analyzed by SPR on a Biacore T200 instrument (GE Healthcare). A research-grade CM5 Biacore sensor chip (GE Healthcare) was activated by a short treatment with N-hydroxysuccinimide/N-ethyl-N’-(3-dimethylaminopropyl) carbodiimide hydrochloride, followed by immobilization of antigen IFNγR at a surface density of approximately 200 RU. The activated groups on the surface of the sensor were subsequently blocked by injecting 1 M ethanolamine as previously described^30^. The kinetic data of the binding A6 scFv to the IFNγR were obtained by injecting increasing concentrations of A6 scFv over the sensor chip at a flow rate of 30 µl/min. The measurements were carried out in PBS containing 0.005% (v/v) Tween-20. Contact time and dissociation time were 1 min and 5 min, respectively. Data analysis was performed with the BIAevaluation software (GE Healthcare). Association (*k*_on_) and dissociation (*k*_off_) rate constants were calculated by a global fitting analysis assuming a Langmuir binding model and a stoichiometry of (1:1). The dissociation constant (*K*_D_) was determined from the ratio of the rate constants as previously described^31^:

$${K}_{{\rm{D}}}={k}_{{\rm{off}}}/{k}_{{\rm{on}}}$$

Enthalpy (Δ*H*) and entropy (Δ*S*) were calculated from the slope and intercept, respectively, of the plot of temperature versus dissociation constant using the van’t Hoff approximation^32^:

$${\rm{l}}{\rm{n}}\,{K}_{{\rm{D}}}={\rm{\Delta }}H/RT\mbox{--}{\rm{\Delta }}S/R$$

where *R* in the gas constant and *T* is the absolute temperature.

### Circular dichroism spectra

CD spectra were recorded on a model J-820 CD spectrometer (JASCO). Far-UV CD measurements were performed with 0.5 mg/mL of IFNγR in PBS using a 1-mm cell and a bandwidth of 1 nm. Spectra were recorded five times for each sample.

### Differential scanning calorimetry

DSC measurements of samples prepared in PBS were carried out on a MicroCal VP-Capillary DSC (Malvern Instruments) at a heating rate of 60 °C per h. To evaluate thermal stability, the temperature where heat capacity was maximum was determined as apparent *T*_m_ (*T*_m,app_) using the software MicroCal Origin 7 (Malvern).

### Accessible surface area and surface charge calculations

Accessible surface area of the antigen was calculated with the Protein Interfaces, Surfaces and Assemblies (PISA) server^33^. Surface charges were calculated using the protein contact potential function in PyMOL^34^.

### Molecular dynamics simulations

MD simulations of wild-type and mutant complexes were performed using GROMACS 2016.3^35^ with the CHARMM36m force field and the CMAP correction^36,37^. Using the CHARMM-GUI^38^, the Fv regions of the structure of the IFNγR-A6 complex (PDB ID: 1JRH) was solvated with TIP3P water in a rectangular box such that the minimum distance to the edge of the box was 15 Å under periodic boundary conditions. Na and Cl ions were added to neutralize the protein charge, then further ions were added to mimic a salt solution concentration of 0.14 M. Each system was energy-minimized for 5000 steps and equilibrated with NVT ensemble at 298 K for 1 ns. Further production run was performed for 100 ns with NPT ensemble. A cutoff distance of 12 Å for Coulomb and van der Waals interactions was used. Long-range electrostatics were evaluated through the Particle Mesh Ewald method^39^. LINCS algorithm^40^ was employed to constrain bonds involving hydrogen atoms. The time step was set to 2 fs throughout the simulations. A simulation was repeated 3 times for each system, and the snapshots were saved every 10 ps. PyMOL and UCSF Chimera^41^ were employed to analyze and visualize the MD trajectories and to render the molecular graphics. Interaction energy was defined as the sum of van der Waals and Coulomb interactions, which were calculated through GROMACS based on the last 80 ns of each trajectory.

## Supplementary information

Supplementary Information

## Data Availability

The datasets generated during and/or analyzed during the current study are available from the corresponding author on reasonable request.
