# Supplementary material for: Exploring designability of electrostatic complementarity at an antigen-antibody interface directed by mutagenesis, biophysical analysis, and molecular dynamics simulations
Source: Sci Rep. 2019 Mar 14;9:4482. doi: 10.1038/s41598-019-40461-5 (PMC6418251; doi:10.1038/s41598-019-40461-5)
Supplement: Supplementary file 1 — Supplementary Information [file 41598_2019_40461_MOESM1_ESM.pdf]

Supplementary Information

Exploring designability of electrostatic complementarity at an antigen-antibody interface directed by mutagenesis, biophysical analysis, and molecular dynamics simulations

Kouhei Yoshida[1], Daisuke Kuroda[1,2], Masato Kiyoshi[3], Makoto Nakakido[1], Satoru Nagatoishi[1,3], Shinji Soga[4], Hiroki Shirai[4], and Kouhei Tsumoto[1,2,3,*]

Table of contents

Figure S1. CD spectra of the antigens and the antibodies.
Figure S2. Molecular surface charge and accessible surface areas of mutated antigen residues.
Figure S3. Transition of Cα-RMSD of the antigen-antibody complexes.
Figure S4. Molecular details of the antigen-antibody complexes during the simulation.
Figure S5. Hypothesis for obtaining better antibodies by modification of antigens.

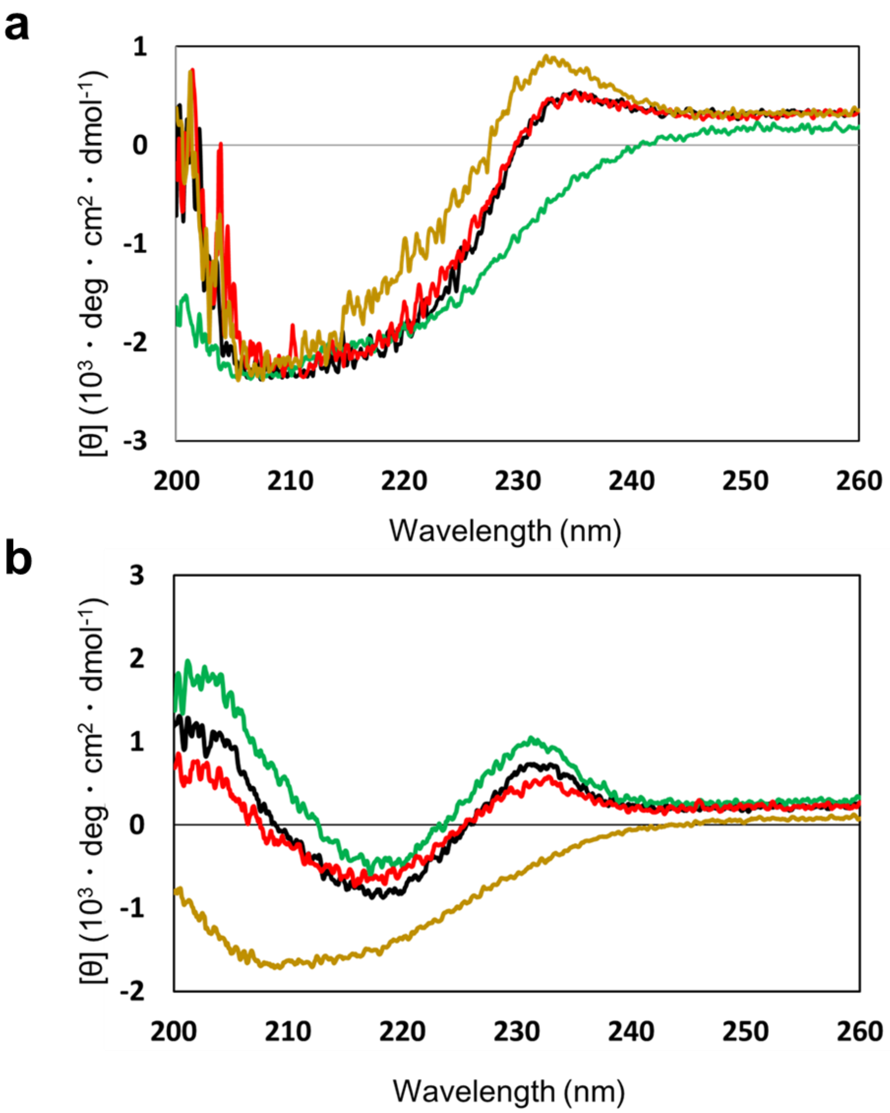

Figure S1. CD spectra of the antigens and the antibodies.

(a) Circular dichroism spectra of the antigens **Ag:WT** (black), **Ag:R84E** (green), **Ag:K98D** (red), and **Ag:K52D** (orange). (b) Circular dichroism spectra of the antibodies **Ab:WT** (black), **V$_L$:E27R** (green), **V$_L$:D28K** (red), and **V$_H$:D54K/D56K** (orange). Each spectrum is the average of accumulations of five measurements.

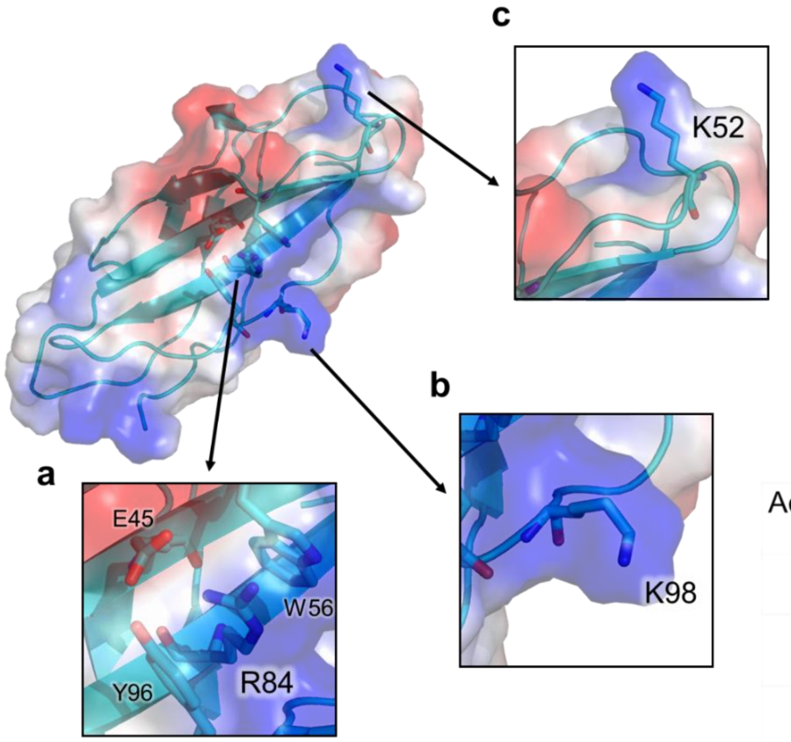

Accessible Surface Area (Å^2)	
K52	134.7
R84	39.3
K98	166.2

Figure S2. Molecular surface charge and accessible surface areas of mutated antigen residues.

The molecular surface charge of the wild-type antigen. Positive charge is indicated in blue, and negative charge is indicated in red. Regions near residues (a) R84, (b) K98, and (c) K52 are magnified, and calculated accessible surface areas are listed.

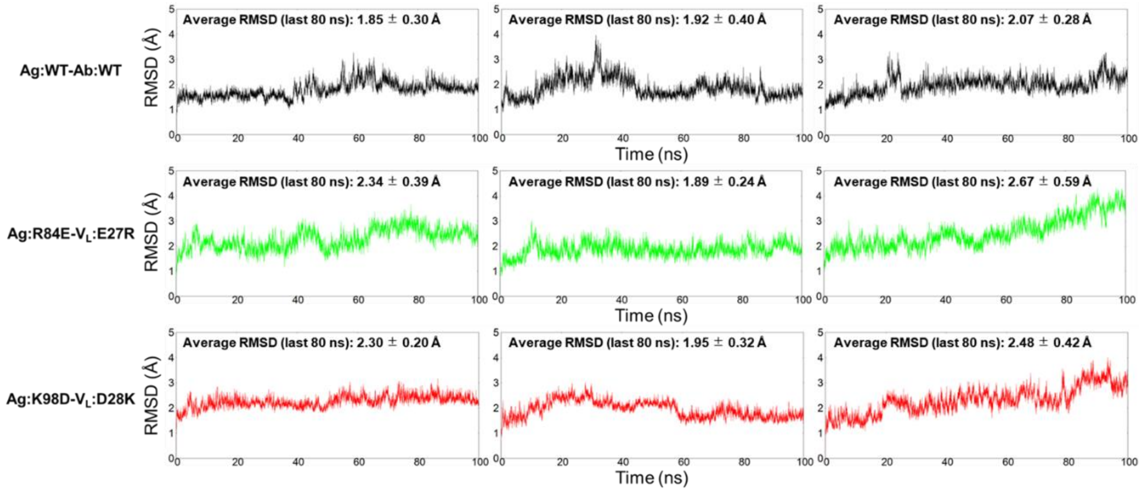

Figure S3. Transition of Cα-RMSD of the antigen-antibody complexes.

Each 100 ns run was performed three times. Averages and standard deviations of the Cα-RMSDs are indicated in figures.

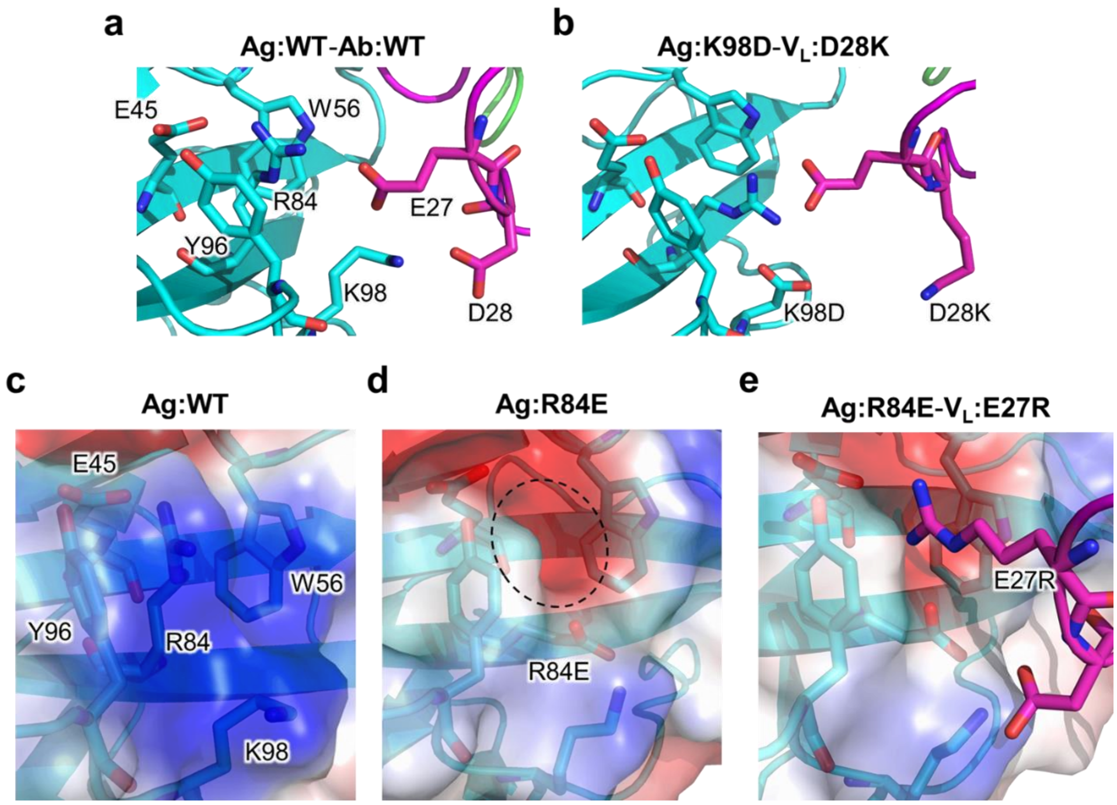

Figure S4. Molecular details of the antigen-antibody complexes during the simulation.

Surrounding residues of the electrostatic interaction at the interface of (a) **Ag:WT-Ab:WT**, and (b) **Ag:K98D-V_L:D28K**. Molecular surfaces based on a snapshot of the simulations around R84 of (c) **Ag:WT** antigen and (d) **Ag:R84E** in the unbound state. The cavity observed in the Ag:R84E but not in the wild-type antigen is indicated with a dashed circle. (e) V_L:E27R being buried in the cavity.

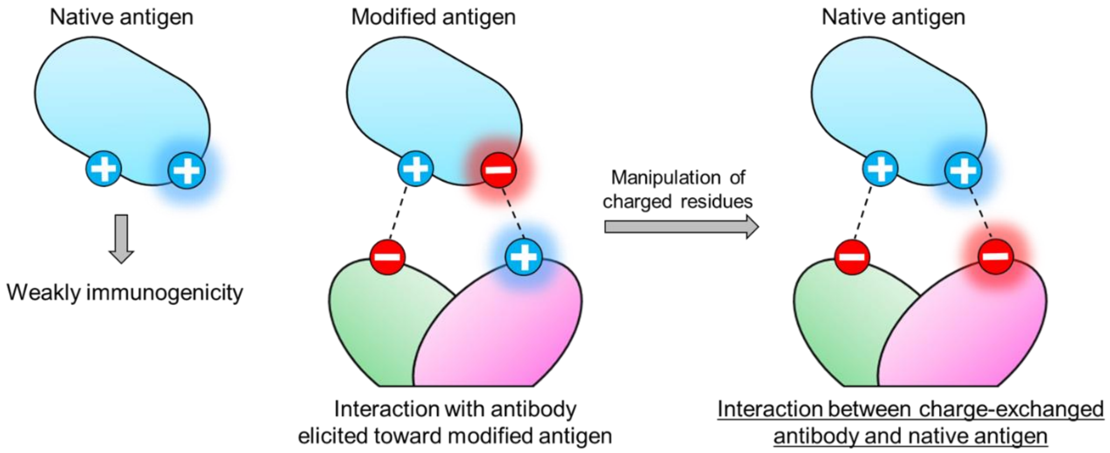

Figure S5. Hypothesis for obtaining better antibodies by modification of antigens.
